# Hypoxia Pathway Proteins and Their Impact on the Blood Vasculature

**DOI:** 10.3390/ijms22179191

**Published:** 2021-08-25

**Authors:** Diego Rodriguez, Deepika Watts, Diana Gaete, Sundary Sormendi, Ben Wielockx

**Affiliations:** Institute of Clinical Chemistry and Laboratory Medicine, Technische Universität Dresden, 01307 Dresden, Germany; diego.rodriguez@mailbox.tu-dresden.de (D.R.); deepika.watts@ukdd.de (D.W.); Diana.Gaete@ukdd.de (D.G.); sundary.sormendi@ukdd.de (S.S.)

**Keywords:** angiogenesis, hypoxia, HIF

## Abstract

Every cell in the body requires oxygen for its functioning, in virtually every animal, and a tightly regulated system that balances oxygen supply and demand is therefore fundamental. The vascular network is one of the first systems to sense oxygen, and deprived oxygen (hypoxia) conditions automatically lead to a cascade of cellular signals that serve to circumvent the negative effects of hypoxia, such as angiogenesis associated with inflammation, tumor development, or vascular disorders. This vascular signaling is driven by central transcription factors, namely the hypoxia inducible factors (HIFs), which determine the expression of a growing number of genes in endothelial cells and pericytes. HIF functions are tightly regulated by oxygen sensors known as the HIF-prolyl hydroxylase domain proteins (PHDs), which are enzymes that hydroxylate HIFs for eventual proteasomal degradation. HIFs, as well as PHDs, represent attractive therapeutic targets under various pathological settings, including those involving vascular (dys)function. We focus on the characteristics and mechanisms by which vascular cells respond to hypoxia under a variety of conditions.

## 1. Introduction

**Figure 1:**
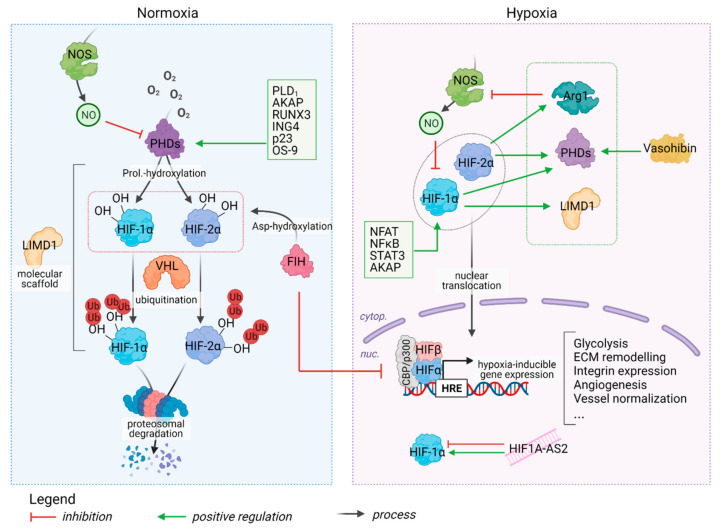
Hypoxia pathway proteins–a regulatory network. Under normoxic conditions, PHDs 1–3 can preferentially hydroxylate HIF1α or 2α, which leads to their VHL-mediated degradation. FIH preferentially hydroxylates HIF1α rather than HIF2α, preventing necessary interaction with co-factors [6,8]. Additionally, LIMD1, a scaffold protein that helps assemble the complex with VHL [9,10] and numerous other regulators of PHD expression/activity have been described (green box—Normoxia [11,12,13,14,15,16,17]). During hypoxia-associated events, including inflammation or tumor growth, HIF1 is activated (green box—Hypoxia), on the other hand, HIF1 supports the transcription of PHD, LIMD1, and Nitric Oxide synthase (NOS), which in turn inhibit HIF1 activity. HIF2 can induce the expression of Arginase 1, which competes with NOS for the same substrate, thus reducing NO production efficiency and reducing HIF1 inactivation as well [9,10,18,19,20,21]. HIF1α-AS2 promotes HIF1 transcription only in the first few hours after hypoxia induction but subsequently inhibits HIF1 activity; in contrast, HIF1α-AS2 continues to promote HIF2 expression even after several hours of hypoxia [4,22].

Maintaining oxygen (O_2_) homeostasis is crucial for the survival of many species and the principal transcription factors that regulate the cellular response to low O_2_ tension, or hypoxia, are the hypoxia-inducible factors (HIFs). Multiple HIF isoforms exist but HIF1α and HIF2α are the most studied, and both bind to HIFβ to form the functional heterodimer. Whilst HIFβ is expressed constitutively, HIFα protein expression is regulated by HIF-prolyl hydroxylase domain (PHD) enzymes and the factor inhibiting HIF (FIH). Specifically, PHDs hydroxylate HIFα subunits at two specific proline residues in an oxygen- and iron-dependent manner; this promotes their ubiquitination by the VHL (von-Hippel–Lindau) enzyme and ultimate degradation by the 26S proteasome. As PHDs require oxygen for catalytic action, they are rendered inactive under hypoxic conditions, which allows HIFα stabilization and its translocation into the nucleus, where it interacts with CBP/p300 and HIFβ, and binds to hypoxia responsive elements (HREs) in target genes that typically promote greater transcription. Of note, even though HIF1 and HIF2 share several target genes, they also have unique binding profiles. For example, whereas HIF1 is mainly linked to regulation of metabolic programming, HIF2 modulates angiogenic extracellular signaling, guidance cues, and extracellular matrix (ECM) remodeling factors [1,2]. Further, their protein levels peak at different times during hypoxia, e.g., while the HIF1 maximum appears at around 12 h followed by a gradual decrease, HIF2 shows delayed induction followed by a stable plateau; thus, HIF2 might be better associated with chronic hypoxia. In contrast to PHDs, FIH hydroxylates HIFα at an asparagine residue, which also prevents its interaction with HIFβ and other factors (e.g., CBP/p300) and inhibits subsequent transcriptional activity [3,4,5]. To date, three main PHD isoforms have been identified, namely, PHDs 1–3; they are expressed in distinct tissues, have specific subcellular localization patterns, and preferentially bind to specific HIFα subunits. Further, deletion of each PHD results in characteristic developmental phenotypes, and as only systemic PHD2 deletion is embryonic lethal, it is arguably the most prominent oxygen sensor. Interestingly, the criticality of oxygen sensing is underscored by the fact that PHD2 transcription itself is also induced by HIF1, which not only ensures swift removal of HIF after sufficient oxygenation has been restored [3,6] but also creates a feedback loop that allows tight regulation of genes participating in the hypoxia response. PHDs also display HIF-independent effects, e.g., whilst PHD1 can hydroxylate IKKβ and thus modulate the activity of nuclear factor kappa B (NFκB), a major inflammatory regulator, PHD2 can hydroxylate eukaryotic elongation factor 2 kinase (eEF2K), which controls protein synthesis [6]. Furthermore, even though combined genomic data analysis using mathematical modelling has identified 6000 hypoxia responsive genes, about 70% of these had no HRE in their proximal promoter [7], suggesting that most of the effects of hypoxia are either HIF-independent or are indirectly targeted by HIFs (see Figure 1).

Endothelial cells (ECs) are a very central cell type in virtually every organ of the body as they are in direct contact with the blood stream; thus, it is intuitive that they are also very responsive to hypoxia, and hence HIFs, in which capacity they regulate vital processes, including cell survival, growth, cell invasion, and energy metabolism. Specifically, while ECs exposed to chronic hypoxia pivot towards increased glycolysis, biosynthesis of amino acids, carbon metabolism, pentose phosphate pathway, fructose/mannose, and cysteine/methionine metabolism [23], those subjected to acute hypoxia exhibit upregulation of genes involved in pyruvate metabolism and glucose transport, suggesting higher occurrence of glycolysis in hypoxic ECs [24]. Besides these direct effects of hypoxia on ECs, many indirect results, such as lactate accumulation in the tumor environment, have also been documented, and accruing evidence suggests that glycolytic activity in ECs, along with metabolism in general, might drive angiogenesis [25,26,27]. The purpose of this review is to discuss our current understanding of the role of hypoxia pathway proteins (HPPs) during angiogenesis in both physiological as well as pathological conditions, with a particular focus on cancer. Additionally, we address present therapeutic strategies targeting HPPs, bringing special attention to the intricate regulatory mechanisms of the HPPs and their context-dependent role.

## 2. Cardiovascular System Formation in the Embryo

Vasculogenesis is the de novo creation of blood vessels by migration and differentiation of endothelial progenitor cells, or angioblasts; in contrast, angiogenesis refers to the formation of new blood vessels that branch out from pre-existing vessels through sprouting and intussusceptive microvascular growth [28]. During embryogenesis, vasculogenesis generates new blood vessels but their remodeling occurs through angiogenesis [29,30]; consequently, angiogenesis is more prevalent in adults than vasculogenesis. 

During embryonic vasculogenesis, hemangioblasts migrate to the yolk sac, where they eventually associate with each other to form blood islands and differentiate into endothelial cells, hematopoietic cells, and vascular smooth muscle cells (SMCs). Hemangioblasts on the outer regions of the blood islands are called angioblasts or precursors of endothelial cells. Fibroblast growth factor 2 (FGF-2) is a major regulator of both vasculogenesis and angiogenesis, and studies in avian embryo have implicated a role for FGF-2 during angioblast differentiation [31,32,33]. Interestingly, HIF1α regulates FGF2 protein levels [34,35] and vice versa, creating a feedback loop [34]. In mouse embryos, FGF-2 induces the expression of vascular endothelial growth factor (VEGF) receptor-2 (VEGFR-2, also known as Flk1), the earliest known receptor for VEGF and a well-characterized activator of angiogenesis [36]. VEGF family members act as chemoattractants for angioblasts, regulate cell proliferation, tube formation, and vessel branching, and are endothelial cell-survival factors; VEGF-A influences angioblast differentiation and initiates a series of steps that lead to a mature vascular network [37]. Hypoxia plays a role in the regulation of this pathway, as it can differentially induce or repress the expression of VEGF family members and their receptors, depending on cell type [38,39,40].

Rapid growth of the embryo during development consumes large amounts of oxygen, leading to hypoxia and increased HIF expression. Thus, in mice while HIF1α expression is high at E8.5 and increases between E9.5 to E18, HIFβ expression varies by less than twofold during E8.5 to E18 [41]. Further, as HIF1α and HIF2α require HIFβ to form their respective functional complexes, deletion of HIFβ dampens the transcriptional activity of both proteins and produces a wide array of phenotypes, including hematopoietic abnormalities and death due to vascular defects [42]. Additionally, embryoid body (EB) cells show lower VEGF and erythropoietin (EPO) expression—these factors are targets of HIF1α that play a crucial role in vasculogenesis and angiogenesis. Additionally, these HIFβ deletion mutants have fewer hematopoietic stem and progenitor cells (HSPCs) at E8.5 and E9.5 and display greater apoptosis in hematopoietic cells. Interestingly, this last phenotype could be rescued by the administration of VEGF [43]. Vessels in the HIFβ^−/−^ embryos were also disorganized, especially in the para-aortic splanchnopleura (pSp)/aorta-gonad-mesonephros region, and HIFβ deletion in pSp cultures inhibited both vasculogenesis and angiogenesis. This phenotype was rescued by adding Sca-1^+^ hematopoietic cells or VEGF to these cultures, suggesting that HIFs coordinate early EC emergence and vessel development by regulating hematopoietic cell survival and production of growth factors by these cells [43].

All solid organs contain monocyte-derived cells that appear early in organogenesis, and for many organs, these cells are critical for normal development. For example, mouse embryonic macrophages were found to have pro-proliferative gene expression signatures, irrespective of their tissue of origin, linking them to tumor-associated macrophages [44]. Macrophages can be found between days 8 and 9.5 post-coitum in both extra-embryonic and embryonic tissues, where they associate with angiogenic tip cells; notably, ablation of these macrophages resulted in fewer vessel intersections in the hindbrain [45]. Moreover, HIF1 deficient zebrafish display impaired macrophage mobilization from the aorta-gonad-mesonephros (AGM) region [46]. 

As with HIFβ deletion mutants, heterozygous loss of HIF2α is embryonic lethal in most mice due to cardiac, vascular, and neural malformations, and mice that grow to adulthood exhibit hypocellularity in the bone marrow, fewer red and white blood cells [47], reduced myeloid multi-lineage progenitors, committed erythroid progenitors, and hemoglobin content in erythroid colonies [48,49,50]. Likewise, specific deletion of HIF1α in embryonic cells expressing VE-cadherin led to a deficiency of HSPCs in the aorta and the placenta [51], but the effects of deleting either HIF1α or HIF2α in the placenta were less severe than homozygous deletions or absence of HIFβ [52]. Furthermore, hypoxic cells expressing high levels of HIF-1α were present in the placenta, while HIF2α expression was mainly found in the decidua. Notably, no significant tissue hypoxia was detected in the placental labyrinth where eNOS was upregulated, but inhibition of NOS resulted in ubiquitous placental hypoxia [53]. Thus, hypoxia appears to affect placental vascularization through tight regulation of HIF1α, HIF2α, and HIFβ. 

## 3. Structure of a Blood Vessel

There are three major types of blood vessels, viz., arteries that carry blood away from the heart, the branching and merging capillaries, and veins that return deoxygenated blood back to the heart. The walls of both arteries and veins consist of three layers: (1) the tunica intima, which is the innermost layer that is in contact with the lumen, and hence, blood flow; (2) the tunica media, an intermediate layer; and (3) the tunica adventitia (or Externa), the outermost layer. The tunica intima is attached to a basal lamina and consists of a single layer of ECs that is in direct contact with the blood stream. The tunica media mostly consists of elastin and smooth muscle cells (SMCs), while the tunica externa consists of a collagen-rich ECM produced by myofibroblasts. Mature blood vessels contain resident progenitor cells capable of differentiating into SMCs that repopulate the tunica media and the tunica intima. They can also produce a unique ECM that varies during vessel maturation, which is responsible for the elasticity and other mechanical properties of these vessels. Notably, hypoxia regulates the expression of many growth factors and pro-enzymes present in the ECM, some of which are also implicated in the degradation of the ECM. Specifically, HIF1α can induce the expression of matrix metalloproteases (MMP) -2, -9, and -15, whereas HIF2α can induce MMP14, and hypoxic environments can downregulate tissue inhibitors of metalloproteases (TIMP) -2 and -3, which generally function as inhibitors of MMPs. Thus, HIFs modulate many characteristics of the ECM, including composition, posttranslational modifications, and rearrangements [54,55,56].

As one of the major functions of blood vessels is to transport oxygen that is bound to red blood cells to different parts of the body, insufficient oxygen pressure induces vasodilation to promote blood flow and thereby enhance tissue oxygenation. This corrective mechanism is partially dependent on hypoxia-induced upregulation of endothelial nitric oxide synthase (eNOS), and the consequent increase in NO production relaxes the smooth muscle cells, leading to vasodilation and increased blood flow [27,57]. ECs can interact with the surrounding ECM through integrins, which have multiple ligands. Sites where integrins are clustered can act as focal adhesion sites between the ECM and various cell types, including platelets, endothelial cells, leukocytes, and smooth muscle cells. Although integrins mainly bind to components in the ECM and to cell-surface ligands, they can also bind to some soluble ligands, such as chemokines or cytokines [58,59]. As the expression of numerous integrins is controlled by the hypoxia pathway, conceivably, variations in oxygen levels can modulate cell–cell and ECM–cell interactions. For example, HIF can regulate integrin alpha IIb beta 3 expression in platelets, leading to altered platelet aggregation under hypoxic conditions [60]. Furthermore, variations in oxygen can also alter the production of red blood cells. During homeostasis in adults, erythropoiesis takes place in the bone marrow. This process is controlled by erythropoietin, which is strictly regulated by changes in oxygen partial pressure and HIFs. A more in-depth review of this process was recently published by our research group [61].

## 4. Angiogenesis

Angiogenesis typically starts in response to the presence of angiogenic cytokines, most noticeably VEGF, and many factors can induce the release of these cytokines, including wounding, ischemia, and hypoxia [62]. Angiogenesis is a complex process and many of the associated factors can play multiple distinct roles, depending on the context in which they are released. Moreover, while cytokines are primary effectors, multiple receptor families also regulate guidance during vascular morphogenesis and are often driven by hypoxia (see Table 1), thereby underscoring the importance of hypoxia as a factor that regulates vascular patterning [63,64]. Therefore, several major proteins involved in angiogenesis initiation are regulated by HIF1, HIF2, or both wherein, while deletion of HIF1α results in inhibition of angiogenesis, absence of HIF2α results in increased angiogenic sprouting but reduced perfusion [23,65,66]. Some of the key ligand–receptor couples involved in the regulation of angiogenesis are VEGFA/VEGFR, Dll4/Notch, ANG2/Tie2, Wnt/Frizzled, PDGF/PDGFR, and ECM/integrins [67].

**Figure 2:**
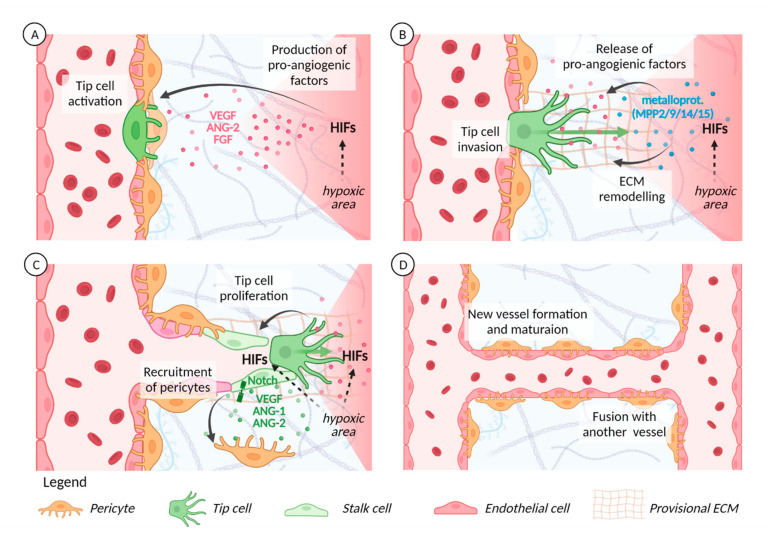
Sprouting angiogenesis is related to hypoxic signaling: (**A**) Cells under hypoxic conditions release angiogenic factors that help loosen the pericytes, resulting in unstable vessels and activation of VEGFR2-expressing tip cells. (**B**) Pericytes detach, EC becomes hyper-permeable in response to VEGF [37], metalloproteases extravasate to degrade the ECM causing release of stored growth factors and proenzymes. A new, provisional ECM is made. Endothelial cells start invading the provisional ECM [116]. (**C**) Hypoxia establishes Tip/Stalk cells. Tip cells are highly mobile, while stalk cells are immotile and proliferative, enabling pericyte recruitment while the branch extends towards hypoxic signals [117]. Pericyte recruitment stabilizes the vessel. Furthermore, cells in the termination area release Vasohibin1, leading to angiogenesis inhibition and vessel stabilization [112]. (**D**) The angiogenic vessel fuses with another vessel. Oxygenated blood circulates through the lumen, dampening the hypoxic signals. Mesenchymal cells migrate along the vessel and form mature pericytes, stabilizing the vessel and helping it mature [109].

An excellent and well-described example of hypoxia-driven angiogenesis pertains to the retina of mice in the first weeks after birth. In the initial step of sprouting angiogenesis, hypoxic astrocytes in the regions of the retina that show no vascularization express VEGF, which then binds to VEGFR2 on endothelial tip cells [55]. Concurrently, ANG2/TIE2 interaction eases the vessel wall [115], reducing endothelial cell–vascular smooth muscle cell interactions (EC-SMC). The ECs then become activated in response to angiogenic signals that are released in response to hypoxia, including VEGF, ANG2, and FGF (see Figure 2). Hypoxia also induces the expression of metalloproteinases that can cause degradation of the existing ECM, which in turn undergoes structural modifications to morph into a provisional ECM; importantly, these changes lead to the release of several growth factors and proenzymes embedded in the ECM [54,55]. 

Next, endothelial stalk cells proliferate and elongate the new branch while trailing behind the tip cell; they also establish tight and adherent junctions, ensuring structural integrity of the new sprouts (Figure 2). Tip cells lead the new branch from the front and extend the long and dynamic actin-based filopodia in response to VEGF, sensing the hypoxic microenvironment and the VEGF gradient through their filopodia and migrating towards the hypoxic areas [118,119,120]. VEGF and hypoxia promote the expression of Dll4 in endothelial cell membranes, which regulates tip/stalk specification by binding to Notch1 expressed in stalk cells; this binding then suppresses tip cell fate and expression of Dll4. Notch also regulates VEGFR-2, -3, and the VEGF co-receptor neuropilin 1 (NRP1) and thereby promotes tip/stalk differentiation responses to VEGF and other signals. Notch also decreases cellular migration and enhances pericyte recruitment, consequently helping to stabilize the vessel. HIF1α interacts with the Notch1 intracellular domain and increases Notch target gene expression [121] and, interestingly, restoring the expression of Dll4 rescues most of the effects of deleting HIF2α in ECs in vivo, underscoring the importance of Dll4 as a target of HIF2α [65].

As the branch of the forming vessel extends, the lumen of the new vessel is formed between adjacent endothelial stalk cells [67,122], which allows the flow of oxygenated blood to the tissue via the newly formed vessel. This reduces hypoxia and creates a negative feedback mechanism that then ensures appropriate vascularization. Proper lumen formation requires MMPs and cell–ECM communication, and while it can be accompanied by activation of genes that promote tube regression [123,124], primitive tubes also recruit pericytes and are sequentially covered by an intermediate and a mature ECM, which inhibit regression, stabilize the vessel, and terminate angiogenesis [58,62,125]. Significantly, hypoxia-controlled release of VEGF, ANG1, and ANG2 are some of the main players that regulate pericyte recruitment [126,127,128]. In contrast to the above, exposing brain endothelial cells to several hours of hypoxia disrupts the blood–brain barrier [129], and pericytes or astrocytes cultured under normoxia promote blood–brain barrier stabilization [130,131]. Thus, the precise molecular dance regulating the termination of angiogenesis is still not well understood and the exact roles that HIF1/2 play are still under investigation. 

Signals initiating angiogenesis can also come from multiple cell types, including cells from the immune system, such as macrophages. Specifically, tumor-associated macrophages (TAMs) can induce angiogenesis by secreting VEGF, MMPs, and Wnts. Most pro-angiogenic TAMs express Tie2, which can also be upregulated by hypoxia, and Tie2 has been shown to upregulate pro-angiogenic genes, suggesting that it may be a marker for pro-angiogenic macrophages. Members of the Wnt protein family also regulate diverse biological processes, including cellular proliferation, survival, differentiation, migration, and apoptosis, and can act as potent angiogenic factors. They transduce cellular signals by binding to Fzd receptors and members of the lipoprotein-related protein 5 or 6 (LRP5/6) family. Besides the known direct effects of Wnt proteins on angiogenesis, they may also induce expression of inflammatory cytokines in ECs, which can further amplify the angiogenic signal [45,132]. Furthermore, using the pathological retinal neovascularization model, we showed that hematopoietic cell HIF2α can control FasL protein levels in the retina, regulating apoptosis of endothelial cells and pathological neovascularization [133].

## 5. Maturation of the Blood Vessels

After vessel formation and initiation of blood flow, mesenchymal cells are recruited, which differentiate into SMCs. They then proliferate until wall thickness adequate for providing stability to the vessel has been achieved; thus, the number of SMC layers that will be present in the mature vessel is established early during maturation. Apart from recruitment and differentiation of mural cells, maturation of the vessel depends on the generation of a mature ECM and it is these ECM–mural cell interactions that help stabilize the vessel and suppress angiogenesis. These processes are regulated by several factors, including EC subtype, shear stress, and hypoxia [54,109,111,134]. Specifically, in vivo HIF2α deficiency in ECs results in a variety of phenotypes, including increased vessel permeability, aberrant endothelial cell ultrastructure, and pulmonary hypertension. Moreover, these animals exhibit defective tumor angiogenesis due to increased hypoxic stress and tumor cell apoptosis [69], implicating a role for HIF2α in the regulation of vessel maturation.

Further, ECs secrete Platelet Derived Growth Factor B (PDGFB) during the initial stages of vessel maturation, which facilitates recruitment of mural cell precursors through interaction with the PDGF Receptor β (PDGFRβ) [109,111]. PDGFB is upregulated by HIF1α [2,110], and when added to lung arterial cells, it induces proliferation of smooth muscle cells, promotes the Warburg effect, and activates HIF1α [135]. After recruitment, endothelium-associated mural cell precursors secrete ANG1, while ECs secrete Tie2, and ANG1 binds to Tie2 [136] to promote the formation of cell–cell adhesions and mural cell association with the vessel wall [137]. These events stabilize nascent vessels and render them resistant to leakiness. Notably, in the absence of VEGF (and thus, angiogenic activity), ANG2 can antagonize ANG1, causing vessels to destabilize and regress; however, in the presence of VEGF, which is released in response to hypoxia, ANG2 facilitates a type of vessel destabilization that leads to vascular sprouting rather than regression [109,111]. Of note, hypoxia controls ANG1 and ANG2 expression in a time-dependent manner [68], and besides the interaction between ANG1–Tie2 and EphrinB2 with its receptor EphB4, mural cell precursors also need endothelial contact to adequately differentiate, which requires communication between gap–junction proteins α1 (GJα1) and γ1 (GJγ1), and signal activation via transforming growth factor β1 (TGFβ1) [109,111]. Hypoxia is involved in this process as it regulates the expression of both TGFβ [98,99] and EphrinB2 [89]. 

## 6. The Role of Inflammation in Angiogenesis

As mentioned above, the hypoxia pathway is an essential regulator of angiogenesis during development and in the adult. Therefore, it is not surprising that this pathway also plays an important role during stress situations such as inflammation, and hypoxia pathway proteins have been tested as therapeutic targets for reducing inflammation [138,139]. Indeed, HIFs are involved in crucial immune cell features, including survival and glycolysis in neutrophils, and promoting inflammatory functions of certain innate immune cells such as dendritic cells, mast cells, and macrophages [140]. Furthermore, we have recently described a central role for HIF2α in the migration of neutrophils through very confined microenvironments, both ex vivo and under various inflammatory settings in vivo [141]. Conversely, HIFs suppress the adaptive immune response by promoting the differentiation of regulatory T-cells and negatively regulating CD4^+^ and CD8^+^ T-cells [139]. Correspondingly, strong cross-communication between endothelial and immune cells during inflammation has also been reported with certain immune cells, e.g., neutrophils and monocytes, reported to play essential roles in inflammation-associated angiogenesis; they also constitute important sources of pro-angiogenic factors such as VEGFA, FGF2, and MMP9, and STAT3-mediated signaling, especially during tumor development [142,143]. Additionally, pro-angiogenic properties of mast cells, i.e., through release of multiple factors such as TNF, FGF2, and VEGFA, have also been reported in certain cancers, such as skin and intestinal adenoma [144]. In vitro, activation of eosinophils leads to secretion of pro-angiogenic factors through degranulation [145], and lymphocytes have also been shown to regulate angiogenesis, both directly and indirectly, such as with the release of similar angiogenic factors by B cells, as by myeloid cells [146]. Similarly, regulatory T-cells promote angiogenesis by releasing VEGFA and thereby suppressing IFNγ-expressing effector TH1 cells. Additionally, while T helper 2 (TH2) cells promote differentiation of tumor-infiltrating monocytes and macrophages into pro-angiogenic tumor-associated macrophages (TAMs), Th1 and Cytotoxic T cells secrete interferon-γ (IFNγ), which inhibits tumor angiogenesis by restricting the proliferation of endothelial cells [147]. 

Apart from this cross-communication, inflammation and angiogenesis also converge at hypoxia as they share common mediators. Interestingly, several such genes, namely, IL-1β, COX2, and SDF-1, which are regulated by HIFs, are involved in angiogenesis and their roles in the inflammatory response have been described [148,149,150]. Available data show that hypoxia-mediated regulation of angiogenic genes associated with inflammation is predominantly controlled via the NF-κB pathway. For instance, IL-6, COX-2, tumor necrosis factor alpha (TNF-α), macrophage inflammatory protein 2 (MIP-2), intercellular adhesion molecule (ICAM), IL-1β, and inducible NOS (iNOS) are some pro-angiogenic factors that are also secreted by immune cells upon hypoxia-induced NF-κB signaling [151]. Another common factor involved in both angiogenesis and inflammation is nitric oxide (NO), which stabilizes HIF1α during inflammation, leading to induction of pro-angiogenic factors [152,153]. Reports also suggest that the contribution of NO to direct activation and gene transcription of VEGF may be sourced from SMCs in this particular scenario [154,155]. Additionally, NO production in ECs is tightly controlled by HIF1/2α-dependent iNOS expression, and as NO directly regulates vessel permeability, it represents an important regulator of not only immune cell extravasation during inflammatory response but also tumor cell migration through the endothelial layers during metastatic processes [156]. Thus, as the role of hypoxia in angiogenesis and inflammation is remarkable, it represents a viable therapeutic target during inflammatory processes such as tumor development.

## 7. Hypoxia in the Angiogenic Tumor Environment

Hypoxia is also a characteristic feature of tumor development because tumor vasculature differs from that seen in healthy tissues in that (1) tumor-derived ECs display higher levels of glycolysis and VEGF secretion than normal ECs [27], (2) tumor vessels have a smaller or more compressed lumen, (3) the vessel wall is abnormal with loosely attached mural cells, (4) the ECM is irregular, and (5) the ECs are poorly connected. All of these lead to irregular vascular perfusion that not only affects oxygen and drug delivery but also eventually facilitates metastasis [124]. 

Research into tumor-associated vasculature has opened at least two possible approaches for therapy, namely, (1) starving the tumor using anti-angiogenic treatment and (2) normalizing tumor vasculature. It has been hypothesized that starving the tumor would arrest its growth, render it dormant, and reduce its size. However, the benefits of restricting angiogenesis have remained modest as such pruning of vessels often leads to increased intra-tumoral hypoxia and consequent radio- and chemo-resistance that trigger pathological angiogenesis and inflammation. Given these disadvantages, another approach, viz., normalizing tumor vasculature, has been proposed. This entails maintaining a balance between pro- and anti-angiogenic factors to reduce vascular permeability and improve blood flow and tumor perfusion [157]. As angiogenesis is strongly linked to hypoxia, it may be possible to target hypoxia pathway proteins to establish this delicate balance between pro- and anti-angiogenic factors. In support of this approach, reports show that in vivo endothelial HIF1α or HIF2α deficiency resulted in enhanced tumor necrosis due to lack of nutrients with consequent reduction in tumor growth and number of tumor vessels [66,69]; in contrast, heterozygous deficiency of PHD2 restored tumor oxygenation and endothelial normalization, inhibiting metastasis [158]. 

## 8. Targeting HIF-Mediated Angiogenesis: A Pharmacological Approach

HIF inhibition has emerged as an attractive therapeutic option against cancer that can disrupt the pathological angiogenic switch prompted by tumor cells. Mechanistically, it can involve two major approaches: (1) direct HIF inhibition to sabotage HIF function and stability and, (2) indirect HIF inhibitors that can obstruct upstream or downstream transcriptional capacities [159]. 

Acriflavine (ACF), a direct HIF inhibitor that prevents HIF heterodimerization, has been shown to inhibit tumor growth and vascularization [160], particularly in glioblastoma, as recent studies on the mechanism of brain tumor growth have revealed a clear association between the overexpression of hypoxia-induced genes and greater probability of invasion, higher recurrence, and poorer clinical outcomes. Mangraviti and colleagues (2017) have evaluated the efficacy of ACF against malignant brain cancer using biodegradable polymers for local sustained drug delivery through the blood–brain barrier. Thus, while in vitro ACF treatment reduced hypoxia-induced overexpression of PGK-1 and VEGF in glioma cell lines, in vivo treatments with different ACF/polymer ratios in a preclinical model led to high survival rates in rats with gliosarcoma [161]. Echinomycin, another well-known direct HIF inhibitor, hampers the binding of HIF1α to the HRE sequence in the VEGF promoter [162], and a recent study by Thomas and collaborators (2005) has demonstrated that nano-delivery of echinomycin induces autophagy-mediated death in pancreatic cancer in vivo, wherein syndecan-1-encapsulated echinomycin, a custom-designed tumor-specific delivery method, resulted in significantly greater survival [163].

Indirect HIF inhibition has also shown promise in recent years and the inhibition of heat shock proteins (particularly Hsp90 and Hsp70) has been reported to have a significant impact on the accumulation of HIF1α proteins in the cytosol, which then promotes their activity. These Hsp chaperones bind to HIF1α and blockade VHL-independent proteasome degradation, permitting proper conformational architecture of HIF1α heterodimers and its subsequent interaction with downstream proteins [164]. The Hsp70 inhibitor IDF-11774, approved for clinical trials phase I study by the Korean Food and Drug Administration, has shown significant dose-dependent reduction in VEGF expression in vivo, along with angiogenesis disruption [165]. More recently, AT-533, a novel Hsp90 inhibitor, has been reported to directly impair HIF1α/VEGF/VEGFR-2-mediated angiogenesis in vitro and in breast carcinoma xenografts in vivo [166]. Hsp inhibitors also severely disrupt tumor-associated angiogenesis, presenting potential benefits for targeted cancer therapy. Additionally, a very promising antiangiogenic and antitumor drug, 2-Methoxyestradiol (2ME2, Panzem^®^) NanoCrystal^®^ Dispersion (NCD^®^) can downregulate HIF1α expression by depolarizing cytoplasmatic microtubules that ultimately block HIF1α translation [159,167]. A combination of 2ME2 and bevacizumab (anti-VEGF) has also led to significant tumor reduction in patients with prostate cancer [168].

New molecules that interact with the HIF pathway to disrupt tumor-associated angiogenesis have also been described. For example, Salinomycin, an antimicrobial agent, has shown anticancer potential [169] and more recently, Dewangan and collaborators (2019) have reported that salinomycin suppresses angiogenesis in the 4T1-induced breast carcinoma model [170]. Another study has revealed that SN-38, an active irinotecan metabolite, can inhibit HIF1α expression upon radiation therapy, wherein this inhibition was followed by a reduction in VEGF expression in colorectal cancer cells (HCT116, SW480). Despite such indirect inhibition of HIF1α, the authors also acknowledge that SN-38-induced radiation sensitivity could be achieved by multiple other pathways [171]; nevertheless, these results imply a potential reduction in the harmful effects of increased HIF1α due to radiation therapy. Detailed aspects of clinical development of HIF2α inhibitors, in direct conjunction with VEGF to treat renal cell carcinoma, have been extensively reviewed recently [172]. Although, HIF inhibition seems to be advantageous in cancer therapy, it is important to note that, to date, there are no clinically approved HIF inhibitors. The vital significance of the HIF pathway, accompanied by a nonspecific delivery mechanism, may permit the cautious use of HIF inhibitors. 

Conversely, a majority of tumor types overexpress PHDs, making these oxygen sensors attractive therapeutic targets. For a more elaborated overview on the potential impact of PHD inhibitors for the treatment of cancer we refer to a recent review from our research group [173].

## 9. Conclusions

This review addressed current advances in the biology of hypoxia pathway proteins and their association with components of the vasculature. We have also provided an overview of the development and functional role of the various components and the impact of HIFs. Further understanding of the role of the hypoxia pathway proteins under physiological and pathological settings, in the context of the vasculature, are warranted, and will be of utmost importance in the development of targeted therapies against disorders involving vascular pathologies. In this respect, recent research advocates for the use of combination therapies to target individual insults generated in clinically relevant settings. Nevertheless, additional research is needed to expand our knowledge of the complex mechanisms underlying the effects of hypoxia pathway proteins, namely, HIFs, PHDs, and related genes.

## Figures and Tables

**Table 1:** Overview of hypoxia-related proteins involved in angiogenesis.

Protein	Function	HIF Pathway	Type of Regulation by Hypoxia	Reference
ANG1, ANG2	Cell-cell adhesion	HIF1 for ANG1; HIF1 and HIF2 for ANG2	Up or down	[2,68,69,70,71]
Wnt/Fzd	Angiogenesis initiation Pericyte recruitment Inflammation regulation	HIF1 and HIF2; independent mechanisms	Up or down	[45,72,73,74,75,76,77]
VEGF family	Angiogenesis initiation and patterning. Tip cell formation Vasculature patterning	HIF1 and HIF2	Up	[78,79,80]
Semaphorins	Vasculature patterning	HIF1 for Sema4D	Up	[81,82,83,84]
Neuropilins	Co-receptor for VEGF; binds semaphorins; Vasculature patterning	HIF1 and HIF2	Up or down depending on cell location and type	[81,85,86,87,88]
Ephrins/Eph	Vascular patterning	HIF1	Up	[89,90]
Netrin1/UNC5 and DCC.	Vascular patterning	HIF1	Up	[91,92]
Slits/ROBO	Vascular patterning		Up	[93]
Dll4/NOTCH	Stalk/tip cell specification; decision to sprout or widen a vessel; arterial specification; EC quiescence and survival	HIF2 for Dll4 FIH for Notch	Up	[1,65,94,95,96,97]
Transforming growth factor β (TGFβ)	Regulation of angiogenic factors and EC growth; vessel maturation (including ECM formation and smooth muscle cell recruitment); angiogenesis resolution.	TGFβ1 can inhibit PHD2	Up in most cases but sometimes down	[98,99,100,101]
Bone Morphogenic proteins (BMP)	Increases angiogenesis; apoptosis regulation; Arterial versus venous specification	Indirect: hypoxia inhibits BMP-binding endothelial regulator, which inhibits BMP expression	Up	[102]
TGFβ receptors type I and II	Each of the different members of the TGFβ family binds a different combination of TGFβ type I and II receptors	Indirect: HIF1 mediates increase of EZH2, which inhibits TGFβRII expression	Up of Alk1 but not Alk5 Down of TGFβRII in prostate cancer.	[102,103,104]
TGFβ receptors type III (Betaglycan and Endoglin)	These are accessory receptors for the TGFβ family. They have a more indirect role in TGFβ signal induction than type I or II receptors	HIF1 for Endoglin	Up for Endoglin Down for betaglycan in rat lungs	[105,106]
Cited2	Vascularization of placenta	Cited2 can inhibit HIF-1α signaling	Up	[107,108]
Matrix metalloproteinases	Degradation of the ECM	HIF1 and HIF2	Up	[54,55]
PDGFB/PDGFBR	Recruitment of mural cells	HIF1	Up	[2,109,110,111]
Vasohibin1	Angiogenesis termination Angiogenesis inhibition	Indirect: Hypoxia induces VEGF release, which induces vasohibin expression Vasohibin1 can induce PHD-mediated degradation of HIF1	Up	[112,113,114]
